# Focus on physiotherapy and manual therapy for infants in Norway, a cross-sectional study on referral practice, and planned interventions

**DOI:** 10.1186/s12887-025-05627-3

**Published:** 2025-04-09

**Authors:** Hege Handeland, Kari Anne Indredavik Evensen, Hilde Stendal Robinson

**Affiliations:** 1 Institute of Health and Function, Western Norway University of Applied Sciences, Bergen, Norway; 2 Department of Clinical and Molecular Medicine, Norwegian University of Science and Technology, Trondheim, Norway; 3 Children’s Clinic, St. Olavs Hospital, Trondheim University Hospital, Trondheim, Norway; 4 Department of Rehabilitation Science and Health Technology, Oslo Metropolitan University, Oslo, Norway; 5 Department of Health Sciences, Institute of Health and Society, University of Oslo, P.O.Box 1089, Blindern, Oslo, 0317 Norway

**Keywords:** Physiotherapy, Manual therapy, Newborn babies, Asymmetries, Motor development

## Abstract

**Background:**

The Norwegian health care system has a mandatory program for close and systematically follow-up on all children, starting in early infancy through the Child Health Care Centers in the municipalities. Additionally, some infants are referred to physiotherapists and manual therapists for several reasons. Little is known about who is referring them and the cause for the referral. In Norway, physiotherapists working with infants can be employed in the communities or work in outpatient clinics, both are within the primary health care system. The main purpose of the present study was to explore the referral practice of infants to physiotherapy and compare those treated by physiotherapists and manual therapists in primary health care in Norway. Furthermore, to describe the planned interventions.

**Methods:**

Cross-sectional study including 444 infants (age under 12 months) referred to physiotherapists or manual therapists working in primary health care in Norway.

**Results:**

Median age (range) of the infants was 14 (1, 52) weeks and 344 were born at due date. Most infants examined by a physiotherapist were referred from other health personnel and more of the referrals to manual therapists were from parents due to their concern. Age at examination was between week 1–12 for 42% of the participants. Infants referred for motor development problems were equally distributed between the physiotherapists and manual therapists. All premature infants were referred to the physiotherapists. Concerning the interventions, both physiotherapists and manual therapists planned to use advice, handling, and stimulation. More of the physiotherapists reported to focus on advice related to motor development and the use of prone play.

**Conclusion:**

The infants in Norway are referred to physiotherapists and/or manual therapists for numerous reasons, and the distribution of diagnoses between the therapists seem reasonable. Infants are mostly referred by other health personnel but also because of parents’ own concern. Based on recommendations, some infants with asymmetries should be examined earlier.

**Trial registration:**

ClinicalTrials.gov Identifier NCT03626389. Registered on August 13th, 2018 (retrospectively registered).

## Background

The Norwegian health care system has a mandatory program for close and systematically follow-up on children, starting early in infancy at Child Health Care Centers (CHCCs) and continue through childhood (in school health services) and the youth period (in youth health care centers). The total number of births in Norway each year has been about 55 000 [1]. The program implies that all newborn babies and their parents are called in for several follow-ups, during their first year, at CHCCs in municipalities throughout Norway. The main focus during the first year is on health promotion, growth, motor and psychological development and systematic vaccinations. The CHCCs staff consist mainly of public health nurses, general practitioners and, in some places, physiotherapists. The staff is in close cooperation with health personnel in primary health care outside the CHCCs, including general practitioners, physiotherapists, and manual therapists (physiotherapists with a master’s degree in manual therapy). According to the national guidelines for the CHCCs [2], physiotherapists are required to take an active part in the health promotion for infants and throughout the childhood.

Today’s parents can find information about almost everything concerning infancy, including normal motor development and asymmetries, on the internet and in social media. However, the information is uncontrolled, can be ill advised, and with low scientific standard. Hence, it can increase parents’ anxiety about the child’s development.

Infants may be referred to physiotherapy and manual therapy from other health care personnel or from the parents themselves for a variety of reasons and conditions [3]. Concerns for motor development, asymmetry, asymmetric movements, and orthopedic conditions are common causes for referring children to primary health care physiotherapy [3], but also problems with breast feeding, infants weight gain and sleep problems are among parents’ concerns. Timely referrals of infants with suspected atypical motor development are important as it enables early intervention. Taking advantage of enhanced neuroplasticity and enriched environment has shown to improve the child’s outcome, prevent complications and support the parents [4].

In recent decades, there has been increased focus on the rising prevalence of head and neck asymmetries in infants in Western countries. Previous studies have connected this rise with the recommendations of supine sleeping-position, from the International best practice for preventing “Sudden Infant Death Syndrome” (SIDS) [5]. This recommendation reduced SIDS tremendously, but increased the number of babies with head asymmetries, known as positional plagiocephaly (PP), due to the external pressure to the same spot on the head [6]. Moreover, this advice may also have resulted in parents avoiding the prone-play position being unaware of its importance and benefits for motor development.

Congenital muscular torticollis (CMT) is another common postural deformity, that might lead to PP [7]. CMT is evident at birth or shortly after, with prevalence ranges from 3.9 to 16% [8, 9] and is defined by reduced neck mobility, usually rotation [7, 8, 10]. Infants with CMT have traditionally been referred to physiotherapy for follow-up. There are no consensus concerning diagnostics, type of treatment, and the effect of treatment on infant head and neck asymmetries. Studies have found that early interventions, starting within the first month of life, are beneficial [8, 11, 12] and that early interventions for postural preferences and CMT can lead to full resolutions of the head and neck asymmetries [8]. Hence, the number of physiotherapy sessions can be reduced.

Lately, a broader range of developmental, environmental, and educational factors have been recommended as treatment modalities [8, 10, 11]. This includes parent education with focus on, among others, alternating the infants’ position to encourage head-turning, emphasize the use of prone play and prevent postural preferences [8, 13], stretching [10, 14] and use of helmet therapy [7, 14]. The latter is not used in Norway. Furthermore, it has been recommended to reduce the time in devices that can make children passive, such as baby-seats and baby wraps, for the youngest infants [10, 11].

In Norway it has been some skepticisms from health personnel and public debate concerning the treatment of infants, and especially the use of joint manipulation/mobilization on infants with asymmetries. Both chiropractors and manual therapists have been the targets for the critics. Moreover, previous studies have shown conflicting results concerning joint manipulation and manipulation in combination with other interventions in the treatment of infants [6, 7, 15]. One argument from the manual therapists is that joint manipulation is seldom used on infants. Moreover, infant asymmetry has been a concern among both health professionals and parents in Norway. Little is known about who is seeking treatment, who is referring the infants, the cause of referral or content of planned interventions.

## Methods

In the present cross-sectional study, we focus on physiotherapists and manual therapists in Norway treating infants. Hence, the primary aim is to explore the referral practice of infants to physiotherapy and manual therapy and compare the infants treated by physiotherapists and manual therapists in primary health care in Norway, as well as describe the planned interventions.

This study was part of the FYSIOPRIM (physiotherapy in primary health care) research program in Norway (2010–2020). A collaboration between researchers at the University of Oslo and the Norwegian University for Science and Technology in Trondheim as well as physiotherapists in primary health care in most parts of Norway [16]. FYSIOPRIM was approved by the Regional Committee for Medical and Health Research Ethics in Norway (no. 2013/2030) and the present study with its additional data collections, was covered by an extension of the original approval in February 2016. Data was collected electronically by approximately 25 PTs in Trondheim Municipality and 24 PTs in Bergen Municipality. In addition, data was collected by 12 manual therapists working with infants in primary health care in different municipalities in Norway, most of them in Oslo (capital) area. At the time of the data collection, it was about 5.3 mill citizens in Norway and around 11.500 working physiotherapists, including around 500 manual therapists. Oslo, Bergen, and Trondheim are among Norway’s largest cities.

### Participants

In total, 444 infants aged 0–12 months referred to physiotherapists and manual therapists were included in the study. Median age (range) of the infants at examination was 14 (1–52) weeks and 344 (78%) were born at due date (i.e., after gestation week 37).

### Procedure for recruitment and data collection

Parents consulting the participating physiotherapists/manual therapists with their infant aged under 12 months, were given written and oral information about the study, and asked to participate. All parents signed informed consent before inclusion of the infant in the study. The physiotherapist/manual therapist completed the examination of the infant as usual; no special instructions were given for the present study. They registered data electronically into the study questionnaire after the examination was completed. Hence, all data were collected electronically and stored at Services for Sensitive data (TSD) at the University of Oslo.

## Measures

The following information was collected: the referral source, the cause for referral, data about the pregnancy, and the birth. They registered data about the baby’s sex, maternal age at birth (weeks), age at examination (weeks), diagnoses (free text), observations and response on clinical tests, goal for treatment (free text), if the babies were to be further referred to other health personnel (yes/no) and if they planned to continue the treatment (yes/no). We categorized the referral sources as follows: Personnel at CHCC, Physiotherapist at CHCC, General practitioner, Pediatric physiotherapist, Specialist health care, Parents own initiative. Maternal age at birth was dichotomized: preterm (< 37 GW) yes/no. The cause of referrals was categorized as follows: Motor development, Asymmetries/asymmetric movements, Orthopedic conditions (i.e., foot alignment, congenital foot deformities, hip dysplasia), Neurologic conditions (referred for physiotherapy related to confirmed neurological diagnosis), Premature birth, Restlessness/sleep problems, Breastfeeding problems, Colic, Parental advice, Other. Planned interventions (refers to what was planned concerning treatment) were categorized into “Parental advice, information, guidance”, “Mobilization, oscillation of joints”, “Combined parental advice and mobilization”, “Joint manipulation”, “Handling and stimulation” which refers to teaching the parents how to alternate the infants’ position to prevent postural preferences, to carry and position the baby to promote symmetry and symmetric movements, “Stretching the sternocleidomastoid muscle (SCM)”, “Mobilization and handling”, “Prone play”, “Improve motor development” which refers to guidance to the parents on how to create environments enhancing and stimulating the infants for motor development and spontaneous symmetric movements, “Massage muscle energy techniques”, “Unknown”.

## Statistical analyses

Descriptive data are presented with mean and standard deviation (SD) for normal distributed, and median and range or frequencies (%) for skewed data. To examine the difference between the groups we used Chi-square tests and independent t-tests as appropriate.

All analyses were performed using SPSS version 27. A significance level of *p* < 0.05 was used.

## Results

Out of the 444 infants available for analyses, 244 (55%) were recruited by physiotherapists in Bergen and Trondheim and 196 (44%) by manual therapists specialized in treating infants.

Total number of boys was 257 (58%) and no sex and age difference were found among infants at examination (Table 1).

**Table 1:** Descriptive data on 444 infants treated by physiotherapists (PT) and manual therapists (MT)

Therapist (*n*)	Mean age (SD) in weeks, at examination	Sex, Boy (%)	Born at due date (> GW37), yes (%)	Total (%)
MT (12)	16(11)	116 (59)	177 (90)	196 (44)
PT (49)	17 (10)	141 (59)	167 (67)	248 (52)
Total	16 (10)	257 (59)	344 (77)	444 (100)

Abbreviations: SD, Standard Deviation, GW, Gestation Week, >, more than

Most of the infants (84%) treated by the physiotherapists were referred from other health care personnel working at CHCCs. A total of 54% of all the infants treated by the manual therapists were also referred from CHCC personnel (including physiotherapists) (Table 2). A larger fraction of the infants treated by manual therapists came without being referred by a health care personnel i.e., they came because of parental concern (28% versus 1% respectively) (Table 2).

**Table 2:** Sources of referral to physiotherapists (PT) and manual therapists (MT) for infants aged 0-12 months

Referred from	Personnel at CHCC	PT	GP	Pediatric PT	Parents own initiative	Specialist health care	Missing data	Total (%)
Referred to								
MT	50 (26)	55 (28)	7 (4)	22 (11)	54 (28)	1 (0.5)	7 (4)	196 (44)
PT	208 (84)	3 (1)	7 (3)	2 (0.8)	2 (0.8)	16 (6)	10 (4)	248 (56)
Total	258 (58)	58 (13)	14 (3)	24 (5)	56 (13)	17 (4)	17 (4)	444 (100)

Abbreviations: CHCC, Child Health Care Center, PT, physiotherapist, MT, manual therapist, GP, general practitioner

We also found differences in causes of referrals to physiotherapists and manual therapists. The main differences were that most of the premature babies, all infants referred to physiotherapy related to confirmed neurological diagnoses and all infants referred for parental advice were referred to the physiotherapists (Table 3), while the few infants referred due to colic and breastfeeding problems were referred to manual therapists.

**Table 3:** Cause of referral to physiotherapists (PT) and manual therapists (MT) for infants aged 0-12 months

	Motor development	Asymmetry/asymmetric movements	Orthopedic conditions*	Neurologic conditions**	Premature birth	Restlessness/sleep problems	Breastfeeding problems	Colic	Parental advice	Other	Total
MT	16 (8)	131 (67)	2 (1)	0 (0)	1 (0.5)	27 (14)	6 (3)	9 (5)	0 (0)	4 (2)	196 (44)
PT	17 (7)	140 (56)	4 (16)	7 (28)	28 (11)	0 (0)	0 (0)	0 (0)	24 (10)	28 (11)	248 (56)
Total	33 (7)	271 (61)	6 (1)	7 (1.5)	29 (7)	27 (6)	6 (1)	9 (2)	24 (5)	32 (7)	444 (100)

* Orthopedic conditions: i.e. foot alignment, walking disabilities **Neurological conditions: referred for physiotherapy related to confirmed neurological diagnosis

Abbreviations: MT, manual therapist, PT, physiotherapist

A total of 28 (6%) infants were examined within the first four weeks after birth, 158 (36%) between 5 and 12 weeks and 258 (58%) after 12 weeks. Among the 28 infants examined within the first four weeks, 61% were referred due to asymmetries/asymmetric movements. Among the 271 infants with asymmetries/asymmetric movements 94% were examined between week 5 and one year after birth (Table 4).

**Table 4:** Causes of referrals distributed across age when examined

Cause of referral	Week 1–4 (%)	Week 5–12 *n* (%)	Week 13–52 *n* (%)	Total *n* (%)
Motor development	1 (3)	14 (42)	18 (55)	33 (100)
Asymmetry/asymmetric movements	17 (6)	101 (37)	153 (56)	271 (100)
Orthopedic conditions (i.e. foot alignment, walking disabilities)	1 (17)	2 (33)	3 (50)	6 (100)
Neurologic* conditions	3 (43)	1 (14)	3 (43)	7 (100)
Premature birth	0 (0)	6 (21)	23 (79)	29 (100)
Restlessness/sleep problems	0 (0)	13 (48)	14 (52)	27 (100)
Breastfeeding problems	1 (17)	4 (67)	1 (17)	6 (100)
Colic	1 (11)	8 (89)	0 (0)	9 (100)
Parental advice	1 (4)	1 (4)	22 (92)	24 (100)
Other	4 (13)	7 (22)	21 (65)	32 (100)
Total	28 (6)	158 (36)	258 (58)	444 (100)

* Referred for physiotherapy related to confirmed neurological diagnosis

Concerning planned interventions, the use of manual techniques was mainly reported by manual therapists, but not as single interventions. More often in combination with parental advice and/or handling the infant (Table 5). For two infants, the reported treatment included joint manipulation. Both physiotherapists and manual therapists reported to use handling and stimulation in their treatment of the infants. More of the physiotherapists reported to have a focus on parental advice related to the child’s development including the use of prone play (Table 5)

**Table 5:** Planned interventions by physiotherapists (PT) and manual therapists (MT) for infants aged 0-12 months

	Advice/ information/ guidance	Joint mob	Combined advice/ joint mob	Handling/ stimulation	Stretching	Combined joint mob /handling	Tummy position	Improve motor develop-ment	Massage muscle energy technique	Unknown/ missing	Total
MT	4 (2)	9 (5)	38 (19)	30 (15)	2 (1)	27* (14)	2 (1)	3 (2)	0	81 (41)	196 (44)
PT	5 (2)	0	0	55 (22)	2 (1)	0	116 (47)	35 (14)	11 (4)	24 (10)	248 (56)
Total	9 (2)	9 (2)	38 (9)	85 (19)	4 (1)	27 (6)	118 (27)	38 (9)	11 (2)	105 (24)	444 (100)

Abbreviations: Mob, mobilization, MT, manual therapist, PT, physiotherapist

* For two of these infants the planned intervention was manipulation

For 54% of the infants, it was not planned any follow-up from the actual therapist, for 66% no further referrals to other health practitioners were planned, and for 2% it was planned a referral to specialist health care

## Discussion

In this cross-sectional study of physiotherapy and manual therapy for infants in Norway, no sex and age differences were found between infants referred to physiotherapists and manual therapists. Most of the infants treated by the physiotherapists were referred from other health care personnel working at CHCCs. More infants were referred to manual therapists by parents based on their own concerns. However, we do not know how many referrals from other health care personnel were based on the parent’s concern at routine follow-ups at the CHCCs.

In our study sample it was few infants with orthopedic (foot deformities) and few referred for physiotherapy on confirmed neurological conditions, and most of these infants were referred to physiotherapists. Moreover, it was also few infants with colic and breastfeeding problems, all of these were referred to a manual therapist.

Both the physiotherapists and the manual therapists reported to use handling and stimulation, which is in accordance with recommendations for head and neck asymmetries [8]. Our results showed further that the physiotherapists seemed to focus more on parental advice related to the child’s development and the use of prone play than did the manual therapists. This difference might be caused by the differences in the referred infants. More of the infants referred with need of parental advice as cause of the referral, and all the premature babies were followed-up at the CHCCs and examined by a physiotherapist. Moreover, prone play is important for normal development [17], recommended for both prevention of delayed development [18], as well as for treatment of asymmetries [8, 11]. Prone play is also recommended for infants by the Norwegian Health authorities [2] and should be part of the advice given to parents. Advising parents and promoting symmetrical motor development for infants should be mandatory for all physiotherapist and manual therapists treating infants in Norway [2]. Previous studies also have focused on how personnel in primary care can facilitate the parents’ involvement in a child`s development [11, 19].

Since all the physiotherapists in our study were connected with the CHCCs, this probably explains why they examined more premature infants. These are usually followed up more closely by physiotherapists employed in the municipalities. More of the manual therapists reported to use mobilization techniques together with giving parental advice and this might be because of referral of more infants with asymmetries/asymmetric movements compared with the physiotherapists. Unfortunately, we have no information of the content of the advice. Hence, we cannot exclude that prone play and stimulation of motor development have been part of the advice. Moreover, the Norwegian National guidelines for preterm infants recommend involving physiotherapists in the follow up when there are concerns related to the infant’s development [20].

Most of the infants in our study (94%) was examined after 12 weeks of age, which is understandable for the premature infants, some with hospitalization, and for infants with restlessness/sleep problems. However, for infants with asymmetries the recommendation is to start treatment early, and if possible, within the first month [8, 11, 12, 21]. We do not know why only 6% of the infants referred for asymmetries/asymmetric movements were examined within the first four weeks, and this finding is important to be aware of for both the CHCCs and the clinicians.

For only two infants in our study the manual therapists planned to use joint manipulation during treatment. This is interesting, especially since previous studies have shown conflicting results [6, 7, 15] and this has been the focus in most of the previous mentioned critics in Norwegian media.

A strength in the present study is the data from a large number of infants examined by physiotherapists and manual therapists in several municipalities in Norway. As far as we know, this is the first study on referral practice and planned interventions among infants under 12 months of age, even though Evensen and co-authors have previously reported on profile of children referred to primary health care physiotherapy in Trondheim [3]. One weakness of the present study is the use of open questions about the cause of referral, planned interventions and content of the interventions. Hence, the categorizing of the answers is subjective and might be criticized. However, we reached consensus regarding categorization of answers (in free text) based on expert opinions and previous publication [3]. We have no follow-up data on most of the infants in our study; hence we have neither information whether the planned interventions were followed nor of the results of treatment. Studies of longitudinal design are needed on this matter.

## Conclusion

Infants in Norway are treated by both physiotherapists and manual therapists for numerous reasons, and the distributions of diagnoses seem to be reasonable and somewhat given by the organization of the follow-up program on infants. The infants are mostly referred by other health personnel but also from the parents. A large number of the infants was referred due to asymmetries/asymmetric movements. Based on recommendations of early treatment in such cases, our findings indicate that some of these infants should have been examined earlier. Longitudinal studies are needed to explore more of the actual content of interventions, the numbers of treatment sessions and the results of treatment.

## Data Availability

The data for this study is stored on a research server (TSD) at the University of Oslo. The datasets generated and analysed during the current study are not publicly available due to the informed consent from the participants does not include permission for data to be shared publicly. The data can be available from the corresponding author upon reasonable request.

## Abbreviations

CHCC: Child health care center
CMT: Congenital muscular torticollis
GW: Gestation week
PP: Positional plagiocephaly
SCM: Sternocleidomastoid muscle
SD: Standard deviation
SIDS: Sudden infant death syndrome
TSD: Services for sensitive data
